# Medicinal properties of *Morus alba* for the control of type 2 diabetes mellitus: a systematic review

**DOI:** 10.12688/f1000research.55573.1

**Published:** 2021-10-08

**Authors:** Jorge Guillermo Morales Ramos, Ambrocio Teodoro Esteves Pairazamán, María Ema Soledad Mocarro Willis, Samuel Collantes Santisteban, Emma Caldas Herrera

**Affiliations:** 1 Universidad Señor de Sipán S.A.C., Chiclayo, Perú, Lambayeque, 14001, Peru; 2 Universidad Privada Norbert Wiener, Chiclayo, Perú, Lambayeque, 14001, Peru; 3 Universidad de San Martín de Porres, Chiclayo, Perú, Lambayeque, 14001, Peru

**Keywords:** Type 2 Diabetes Mellitus, Morus alba, T2DM control, blood glucose

## Abstract

**Background:** The objective of this review was to evaluate the medicinal potential of *Morus alba* leaves on the control of type 2 diabetes mellitus (DM2). The research question was: what is the therapeutic potential of *Morus alba* leaves for the control of DM2?

**Methods:** This systematic review was based on the Preferred Reporting Items for Systematic reviews and Meta-Analyses (PRISMA) guidelines. The included studies were extracted from Scopus, Pubmed, ScienceDirect, Scielo, and Google Scholar; January 2015 to July 2021. Key search terms were MeSH and DeCS: *Morus alba*, mulberry, hypoglycemic agent. The inclusion criteria were: studies in rats administered *Morus alba* leaf extracts; studies that included the dimensions of lipidemia and glycemia; studies that included indicators such as fasting glucose, postprandial glucose, glycosylated hemoglobin, triglycerides, low-density lipoproteins, total cholesterol, and insulin resistance. Exclusion criteria: studies in which *Morus alba* leaves were administered with other plants; studies with other parts of the *Morus alba* plant; proteomic studies, cancer, duplicate studies, *in vitro* studies, and evaluation of included studies. All included investigations were evaluated for biases.

**Results:** Of 253 studies found, 29 were included. The extracts of *Morus alba* leaves at the phytochemical level improve glucose uptake. Chlorogenic acid, isoquercitrin, and quercitrin, present in the leaves of *Morus alba*, have hypoglycemic properties and an ameliorating effect on diabetic nephropathy. This leaf has pharmacological effects such as glucose absorption, insulin secretion production, antioxidant and anti-inflammatory agent, antihyperglycemic and antihyperlipidemic activities, and obesity management.

**Conclusions:** *Morus alba* leaves have pharmacological effects on DM2 that include glucose absorption, production of insulin secretion, antioxidant agent, antihyperglycemic and antihyperlipidemic activities, and obesity control. Beyond these results, there is a lack of studies on the potential and synergistic effects of *Morus alba* leaves' components, limiting the possibility of a more effective therapy using the plant's leaves.

## Introduction

The World Health Organization (WHO) in 2014 globally reported that 422 million adults had diabetes. ^1^ Diabetes mellitus (DM) has become a major global problem with a significant health impact because the number of cases is constantly increasing and also because the long-term projections are less and less optimistic, ^2^ becoming one of the diseases selected by the world health authorities that require priority attention. In 2019, DM was the leading cause of 1.5 million deaths, and in 2012 2.2 million people died due to hyperglycemia. ^2^ By 2030, it is estimated that the global prevalence of diabetes will be 10.2% (578 million people) and 10.9% (700 million) by 2045, ^3^ Of the three types of diabetes mellitus, the most common is type 2 (T2DM).

T2DM is frequently linked to obesity. A predominant insulin resistance, accompanied by deficient hormone activity, leads to an increase in its secretion. ^4^ T2DM is a disease associated with multiple factors and of a chronic nature, so its treatment must be seen integrally in the patient's life. Therefore, continuous adjustments are needed according to the specific requirements of each patient. ^5^

An approach to diabetes care has been proposed by Kenneth S. Polonsky ^6^ and is based on the formation of work teams that involve health professionals for patient care and on the development of care delivery models for diseases using this approach, the Diabetes Prevention Program (USA) ^6^ demonstrated that physical exercise and weight loss could reduce the risk of diabetes in predisposed people by 58%. The main effects are also seen after treatment with metformin or pioglitazone.

The WHO, in its latest report on diabetes, points out that there are a variety of antidiabetic pharmacological therapies. However, most of them are expensive and cause adverse effects, impacting health systems, especially diabetic patients. The other problem is that only a minority in developing countries generally have insulin and drugs considered essential in controlling diabetes, such as antihypertensives and lipid-lowering drugs, within reach. Hence, it is necessary to establish regulatory policies and programs to improve equity in access. ^1^

The National Demographic and Family Health Survey (ENDES) carried out in 2013 in Peru indicated a progressive increase in the number of T2DM cases, mainly due to the lifestyle of the Peruvian population. Epidemiological data suggest that there is a prevalence of 33.8% of overweight and 18.3% of obesity. ^7^ In Peru, diabetes affects 7% of the population, representing 31.5% of acute myocardial infarctions and another 25% of cerebrovascular accidents. T2DM is equivalent to 96.8% of outpatient visits with this condition. The prevalence of glucose intolerance is 8.11%, and the variation in fasting glucose is 22.4%. The prevalence data for overweight, MS in adults, and obesity is 34.7%, 25%, and 17.5%, respectively. ^8^

The present review focuses its study on the leaves of the *Morus alba* plant, which is distinguished by being used in traditional medicine and which is also a very promising species. During the last 20 years, the great majority of studies have addressed the isolation, and active principles of the polysaccharides of this plant have focused mainly on its leaves and fruits, which are both medicinal and edible. ^9^ The results of preclinical investigations of the last decade suggest that *Morus alba*, as a therapeutic agent, is of utmost importance for treating DM; the polyhydroxy alkaloids, flavonoids, and polysaccharides of Morus may be; active components of great potentiality. ^10^ In addition, this product, especially in oriental medicine, has been used for fever, liver damage, vision problems, and to lower blood sugar levels. However, the studies carried out to date on the antidiabetic compounds in this product are insufficient. ^11^

Different parts of *Morus alba* are used in the treatment of various diseases, are required for their medicinal properties such as antioxidant, antibacterial, antidiabetic, and antiviral. In addition, it has active components in the leaf, such as polysaccharides, that can lower the glucose level, blood, and blood pressure. The leaves are used for hypoglycemia and also as antioxidants, which contain the chemical components: scopolin, isoquercitrin, astragalin, rose oxide, benzyl glucopyranoside, and phenolic acids such as gallic, protocatechuic, p-hydroxybenzoic, vanillic, chlorogenic, syringic, p-coumaric, ferulic, and m-coumaric, also the polysaccharide deoxynojirimycin (DNJ). ^12^

The present systematic review focuses on the active principles found in the leaves of the *Morus alba* plant and its medicinal properties, taking into account its use in traditional medicine. It is based on the fact that the extracts of *Morus alba* leaves contain flavonoids as main constituents, which also include antioxidant, antidiabetic, antihyperlipidemic, antiobesity, cardioprotective activities, and are rich in anthocyanins and alkaloids. ^13^ The purpose of this systematic review was to evaluate the medicinal potential of *Morus alba* leaves on the control of T2DM. The research question was: what is the medicinal potential of *Morus alba* leaves in the control of T2DM?

## Methods

This review is based on the methodology established in the Preferred Reporting Items for Systematic reviews and Meta-Analyses (PRISMA) statement. (14, 41) All the scientific information included in the study was extracted from international databases such as Scopus, Pubmed, ScienceDirect, Scielo, and Google Scholar from January 2015 - July 2021; for identification, filtering, eligibility, and inclusion, the Mendeley Desktop 1.19.8 web application was used. The protocol of this systematic review was registered in the PROSPERO database, ID: 274164, on 18 ^th^ August, 2021 (pending).

The primary auxiliary strategy (applied to all declared databases) to identify studies that correspond to the research question was the following:

• TITLE-ABS-KEY (morus AND alba) AND PUBYEAR > 2013
• (TITLE-ABS-KEY ( morus AND alba) OR TITLE-ABS-KEY (morus AND alba AND leaves)) AND PUBYEAR > 2013
• (TITLE-ABS-KEY (morus AND alba) AND TITLE-ABS-KEY (mulberry)) AND PUBYEAR > 2013
• (TITLE-ABS-KEY (morus AND alba) AND TITLE-ABS-KEY (mulberry) AND TITLE-ABS-KEY (hypoglycemic AND agent)) AND PUBYEAR > 2013

The auxiliary search strategy (applied to all declared databases) to identify other studies that correspond to the research question was the following:

• (TITLE-ABS-KEY (Diabetes AND mellitus ) AND TITLE-ABS-KEY (type 2 diabetes AND mellitus) AND TITLE-ABS-KEY ( blood AND glucose) AND TITLE-ABS-KEY ( lipid-lowering AND agent ) AND TITLE-ABS-KEY (lipid-lowering AND agent) AND TITLE-ABS-KEY (blood AND lipids) AND TITLE-ABS-KEY (glycemic AND control) AND TITLE-ABS-KEY (postprandial AND hyperglycemia)) AND PUBYEAR > 2013

The inclusion criteria were: studies in rats administered *Morus alba* leaf extracts, studies that included dyslipidemia and glycemic dimensions, studies that included indicators such as fasting glucose, postprandial glucose, glycated hemoglobin, triglycerides, low-density lipoproteins, total cholesterol, and insulin resistance. Only empirical articles, systematic reviews, case studies were taken into account; Due to its importance, a descriptive analysis was included. The exclusion criteria were: studies in which *Morus alba* leaves were administered with other plants, studies with other parts of the *Morus alba* plant (fruits, roots, stems), proteomic analyses, cancer, duplicate studies, inward studies, *in vitro* and evaluation of included studies. Letters to the editor, narrative reviews, or scientific journal editorials were excluded from this study.

To correct biases, the PRISMA 2020 checklist ^14^ was first used. The empirical articles were evaluated through an analytical rubric prepared according to the parameters declared in the SSAHS scale of López-López E, Tobón S, Juárez-Hernández LG) to consider scientific articles. ^15^ Systematic reviews were assessed using the Quality Assessment of Systematic Reviews and Meta-Analyses, using an observation guide (checklist style according to the parameters of National Heart, Lung, and Blood Institute). ^16^ The checklists can be found as extended data. ^41^

Two experienced researchers on the team reviewed the systematic reviews—the original investigations by the three remaining members. The coordination and development of these activities were through the Zoom video chat software. To include the studies, the relationship of each one with the research question was verified, based –fundamentally– on the terms: *Morus alba*; mulberry; and hypoglycemic agent. Then, Quality Assessment of Systematic Reviews and Meta-Analyzes and a scale for evaluating scientific articles were used to guarantee strict compliance with the inclusion and exclusion criteria stated in previous paragraphs. The web application used throughout the identification, screening, eligibility, and inclusion process was Mendeley. To collect the relevant data from each report, PRISMA was used. In the final process of compiling/coding the reports already examined, the entire research team was involved. The variables for which relevant information was sought were diabetes mellitus, type 2 diabetes mellitus, blood glucose, lipid-lowering agent, lipoproteins, blood lipids, glycemic control, postprandial hyperglycemia.

## Results

**Figure 1.**
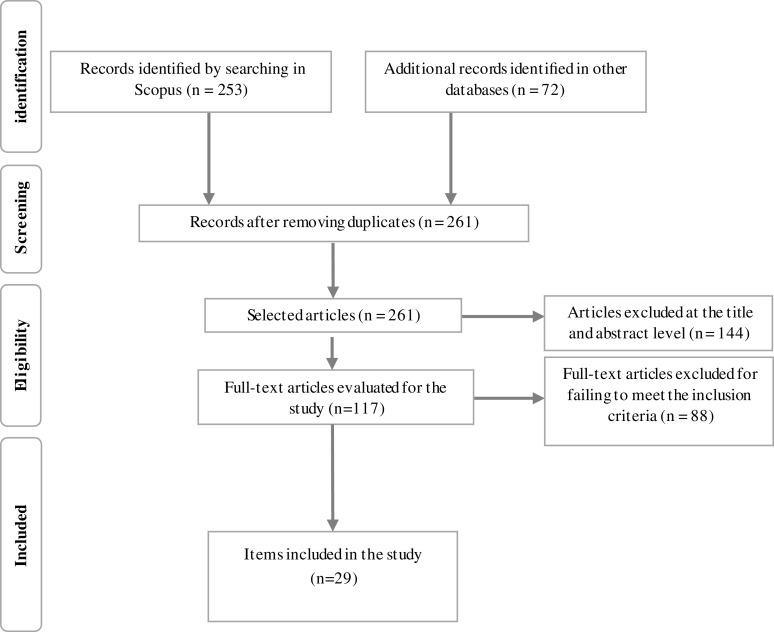
Flow diagram of the identification, screening, eligibility and included articles.

Figure 1 shows that of an initial sample of 261, 29 investigations (17 originals and 12 systematic reviews) were included for 11.11%. The articles rejected for not meeting the inclusion criteria were 232, mainly because they lacked theoretical-methodological support, detected through the quality assessment instruments.

Table 1 shows the distribution of the search results by author, article title, and type of study/characteristics of the studies finally included. 58,62% (original research); 41,38% (systematic reviews). All investigations were included based on the inclusion and exclusion criteria provided in the study methodology.

**Table 1.** Included articles that met the eligibility criteria. OR = original research; SR = systematic review.

No.	Authors	Article title	Type of study/characteristics of the studies
1	Ahn E, Lee J, Jeon Y-H, Choi S-W, Kim E	Antidiabetic effects of mulberry (Morus alba L.) branches and oxyresveratrol in streptozotocin-induced diabetic mice	Original Research (OR): *Morus alba* ethanol extracts were administered orally once daily for 22 days in amounts of 0.5 or 1 g/kg of body weight to a diabetic mouse, induced with streptozotocin.
2	Chan EW-C, Lye P-Y, Wong S-K	Phytochemistry, pharmacology, and clinical trials of Morus alba	Systematic Review (SR): *Morus alba* leaves possess biological activities: antioxidants, antidiabetic, antihyperlipidemic, antiobesity, glycosidase inhibitor, cardioprotective, antimicrobial, and cytotoxic.
3	Díaz SM, Cazaña MY, Pérez HY, Valdivia ÁA, Prieto AM, Lugo MY	Qualitative evaluation of secondary metabolites in extracts of Morus alba L. (Mulberry) varieties and hybrids	OR: The fresh leaves collected from *Morus alba* were washed, disinfected, dried, and pulverized; then, the extracts were obtained with n-hexane, ethanol, and water, which were filtered and subjected to phytochemical identification tests. A toxicity evaluation of the aqueous extracts was carried out in rats to determine their safety.
4	Ge Q, Cheng L, Tang M, Zhang S, Liu L, Gao L, *et al*.	Analysis of mulberry leaf components in the treatment of diabetes using network pharmacology	OR: The study identified 202 compounds of the *Morus alba* leaf using mass spectrophotometry and gas chromatography, 22 components with curative properties were identified on Diabetes Mellitus.
5	Go EJ, Ryu BR, Yang SJ, Baek JS, Ryu SJ, Kim HB, *et al*.	Anti-obesity Effect of the Flavonoid Rich Fraction from Mulberry Leaf Extract	OR: Flavonoid-rich fractions were administered to rats C57BL/6 mice fed a high-fat diet, and their development in obesity was investigated.
6	He X, Fang J, Ruan Y, Wang X, Sun Y, Wu N, *et al*.	Structures, bioactivities and future prospective of polysaccharides from Morus alba (white mulberry): A review	SR: The leaves and fruits are rich in polysaccharides with promising activities: antidiabetic, immunomodulatory, anti-inflammatory, antioxidant, antiobesity, hepatoprotective, and renoprotective.
7	Jeszka-Skowron M, Flaczyk E, Jeszka J, Krejpcio Z, Król E, Buchowski MS	Mulberry leaf extract intake reduces hyperglycaemia in streptozotocin (STZ)-induced diabetic rats fed high-fat diet	OR: A high-fat diet was applied for four weeks to Wistar rats to induce type 2 diabetes. The animals were subsequently treated with extracts of dried leaves of *Morus alba* and ethanol or acetone.
8	Ji S, Zhu C, Gao S, Shao X, Chen X, Zhang H, *et al*.	Morus alba leaves ethanol extract protects pancreatic islet cells against dysfunction and death by inducing autophagy in type 2 diabetes	OR: The main chemical components of the ethanol extract of *Morus alba* leaves were analyzed to determine their identification and quantification using the high-performance liquid chromatography (HPLC) technique. Rats were used in which T2DM was induced using a high-fat diet combined with streptozotocin; the MLE was administered by oral gavage.
9	Jiao Y, Wang X, Jiang X, Kong F, Wang S, Yan C	Antidiabetic effects of Morus alba fruit polysaccharides on high-fat diet- and streptozotocin-induced type 2 diabetes in rats	OR: Rats were induced to have Diabetes on a high-fat, low-dose streptozotocin diet. The animals were treated with two polysaccharide fractions from the *Morus alba* fruit (MFP50 and MFP90). Normal rats and diabetic rats treated with metformin were compared.
10	Kan J, Velliquette RA, Grann K, Burns CR, Scholten J, Tian F, *et al*	A novel botanical formula prevents diabetes by improving insulin resistance	OR: Tests were carried out in male Sprague Dawley rats, administering extracts of a recipe composed of extracts of leaves of *Morus alba*, American ginseng, and Trigonella foenum-graecum, to determine their inhibitory capacity on the enzymes α-amylase and α- glucosidase, in a cell-free system.
11	Kar A, Mukherjee PK, Sankarshan S, Bahadur S, Ahmmed SKM, Subrata P	Possible herb-drug interaction of Morus alba L.- a potential antidiabetic plant from Indian traditional medicine	OR: An assay was carried out to quantify the active compounds of the *Morus alba* leaf extract by the RPHPLC method. The inhibitory potential was determined in pooled CYP450 as well as recombinant CYP450.
12	Khyade VB	Influence of Leaf Decoction of Mulberry, Morus alba (L.) on Streptozotocin Induced Diabetes in Brown Rat, Rattus norvegicus	OR: A test was carried out to evaluate the effects of treatment with the product resulting from the decoction of *Morus alba* leaves on specimens of Rattus norvegicus", for which 20 g/L was administered. One week before treatment, experimental Diabetes was induced by applying streptozotocin.
13	Król E, Jeszka-Skowron M, Krejpcio Z, Flaczyk E, Wójciak RW	The Effects of Supplementary Mulberry Leaf (Morus alba) Extracts on the Trace Element Status (Fe, Zn and Cu) in Relation to Diabetes Management and Antioxidant Indices in Diabetic Rats	OR: Male Wistar rats were induced with Diabetes by placing them on a high-fat diet and administering streptozotocin. 38 rats divided into 5 experimental groups were used: (1) AIN-93M-fed healthy control group; (2) HF control group; (3) HF diabetic group; (4) HF + AE diabetic group (6 g/kg diet); and, (5) HF + EE diabetic group (diet 6 g/kg).
14	Lim SH, Yu JS, Lee HS, Choi C-I, Kim KH	Antidiabetic Flavonoids from Fruits of Morus alba Promoting Insulin-Stimulated Glucose Uptake via Akt and AMP-Activated Protein Kinase Activation in 3T3-L1 Adipocytes	OR: A phytochemical analysis of the *Morus alba* ethanol extract was performed using high-performance liquid chromatography (HPLC). After purification, it was possible to isolate two main active principles: rutin and quercetin-3-O-β-d-glucoside (Q3G), to later apply them to 3T3-L1 adipocytes.
15	Liu C, Xiang W, Yu Y, Shi ZQ, Huang XZ, Xu L.	Comparative analysis of 1-deoxynojirimycin contribution degree to α-glucosidase inhibitory activity and physiological distribution in Morus alba L	OR: 12-day-old male Wanxi white geese were used, which were randomly assigned to 4 treatment groups: (1) Control group with simple diet without 1-deoxynojirimycin (DNJ); (2) Group L-DNJ; (3) Group M-DNJ; and (4) HDNJ Group; these last three groups had complementary elemental diets with DNJ: 0.05 mg/g, 0.1 mg/g, and 0.15 mg/g, respectively. The feeding lasted for six weeks.
16	Mahboubi M	Morus alba (mulberry), a natural potent compound in management of obesity	SR: The objective of this review was to evaluate the potential effect of *Morus alba* as an antiobesity agent; therefore, various databases were searched: PubMed, Science Direct, Springer, Wiley, and Google Scholar) unpublished data (reports from R&D, thesis, and dissertations).
17	Mahesh DS, Vidhathri BS, Vidyashree DN, Narayanaswamy TK, Subbarayappa CT, Muthuraju R	Biochemical Composition and Pharmacological Properties of Mulberry (Morus spp.) - A Review	SR: The biochemical composition and pharmacological properties of various mulberry species are reviewed. It includes its nutritional value, phytochemical composition, antioxidant activity, mineral composition, hypoglycemic activity, antiobesity and hyperlipidemic action, antioxidant, antidiabetic, anti-inflammatory, and antiallergic function, its vasoactive, neuroprotective, renoprotective, and anticancer action.
18	Mellado-Orellana R, Salinas-Lezama E, Sánchez-Herrera D, Guajardo-Lozano J, Díaz-Greene EJ, Rodríguez-Weber FL	Pharmacological treatment of diabetes mellitus type 2 directed to patients with overweight and obesity	SR: The evidence-based recommendations are oriented towards pharmacological and surgical intervention and changes in the lifestyle of obesity management as part of the comprehensive treatment of patients with type 2 diabetes mellitus.
19	Meng Q, Qi X, Chao Y, Chen Q, Cheng P, Yu X, *et al*.	IRS1/PI3K/AKT pathway signal involved in the regulation of glycolipid metabolic abnormalities by Mulberry (Morus alba L.) leaf extracts in 3T3-L1 adipocytes	OR: The *Morus alba* leaf extract was prepared to contain the flavonoids (1 g/ml), and the routine of the flavonoids was determined using the high-performance liquid chromatography (HPLC) technique with the Agilent 1260 system.
20	Meng Q, Qi X, Fu Y, Chen Q, Cheng P, Yu X, *et al*.	Flavonoids extracted from mulberry (Morus alba L.) leaf improve skeletal muscle mitochondrial function by activating AMPK in type 2 diabetes	OR: Skeletal cells were cultured *in vitro*, which were treated with or without flavonoids extracted from *Morus alba.* For in vivo studies, db/db mice were used for type 2 diabetes, with/without MLF therapy. Α-SMA immunofluorescence staining and Coomassie brilliant blue staining were used to identify differentiated L6 cells. The glucose level and the L6 ATP level were carried out by optical density detection, and the viability of the cell was carried out by the MTT method.
21	Rodrigues EL, Marcelino G, Silva GT, Figueiredo PS, Garcez WS, Corsino J, *et al*.	Nutraceutical and Medicinal Potential of the Morus Species in Metabolic Dysfunctions	SR: The genus Morus is used for the treatment and prevention of Diabetes mellitus through its hypoglycemic action, as it has phenolic, anthocyanin, and flavonoid components, in greater or lesser concentration depending on the species; in the case of *Morus alba*, it has low concentrations of flavonoids and anthocyanins.
22	Saeedi P, Petersohn I, Salpea P, Malanda B, Karuranga S, Unwin N, *et al*.	Global and regional diabetes prevalence estimates for 2019 and projections for 2030 and 2045: Results from the International Diabetes Federation Diabetes Atlas, 9th edition	SR: 255 high-quality sources published between 1990 and 2018 from 138 countries were studied. Countries with low-quality data were extrapolated based on geography, ethnicity, language, and economy. A regression was applied to generate estimates taking into account the specific age between 20-79 years and the prevalence of Diabetes.
23	Seclén S	Diabetes mellitus in Peru where we are going	SR: Analyzes epidemiological data extracted from surveys carried out in Peru: ENDES, 2013; ENAHO, 2009-2010; PERUDIAB, 2012; WHO DIAMOND, SEARCH Study.
24	Swathi P, Gana Manjusha K, Vivekanand M, Ramkishan A, Bhavani B	Effect of *morus alba* against hyperglycemic and hyperlipidemic activities in streptozotocin induced diabetic nephropathy	OR: It was applied in male Wistar rats induced to Diabetes with streptozotocin, applying an ethanolic extract of *Morus alba* (AME) leaf. After 12 weeks of starting the experimental work, the following were biochemically analyzed: blood glucose, glycosylated hemoglobin, bilirubin, albumin, creatinine, total protein, urea, lipid profile, and urine. Histopathological observations were also made.
25	Villena JE	Diabetes Mellitus in Peru	SR: It was based on the systematic search of bibliographic sources included in Scielo, PubMed, literature from the World Health Organization, the International Diabetes Federation, and Peruvian agencies related to the subject in question.
26	Wei H, Liu S, Liao Y, Ma C, Wang D, Tong J, *et al*.	A systematic review of the medicinal potential of mulberry in treating diabetes mellitus	SR: *Morus alba* studies are systematized, classifying them by their chemical composition, pharmacological effects of the different parts of the plant on diabetes mellitus, which included: glucose absorption, insulin secretion, oxidative and anti-inflammatory processes.
27	Wen P, Hu TG, Linhardt RJ, Liao ST, Wu H, Zou YX	A review of bioactive compounds and advanced processing technology	SR: A description of mulberry is detailed, examining its main active principles, including polysaccharides, alkaloids, phenols, anthocyanins, and flavonoids. Technological advances for its extraction are described: solid-liquid extraction, supercritical-fluid extraction, liquid-pressurized extraction, microwave-assisted extraction, enzymatic assisted extraction, ultrasound-assisted extraction, solid-phase extraction; other techniques for its separation, such as ion-exchange chromatography, gel filtration chromatography, preparative liquid chromatography, countercurrent chromatography, silica gel chromatography, and macroporous resin adsorption.
28	Wilson RD, Islam MS	Effects of white mulberry (Morus alba) leaf tea investigated in a type 2 diabetes model of rats	OR: It was carried out in male Sprague-Dawley rats of 6 weeks, divided into four groups; They were given high doses (0.5%) and low doses (0.25%) of white mulberry leaf tea. The groups were classified: standard control group (NC), diabetic control group (DBC), high dose diabetic group (DMTH), and low dose diabetic group (DMTL). T2DM was induced with streptozotocin, and four weeks later, analytical tests were performed to evaluate the levels of blood glucose, serum insulin, uric acid, glucose intolerance, glucose intolerance, fructosamine, AST, ALT, albumin, creatinine, and cholesterol.
29	Younus I, Fatima A, Ali SM, Usmani S, Begum Z, Badar S, *et al*.	A review of ethnobotany, phytochemistry, antiviral and cytotoxic/anticancer potential of morus alba linn	SR: The article summarizes the phytochemistry, ethnobotany, antiviral and cytotoxic properties, and anticancer potential of *Morus alba* Research revealed that various parts of the plant contained flavonoids, glycosides, polysaccharides, tannins, and lectins.

Of the set of excluded studies, the proposal by Hwang SH, Li HM, Lim SS, Wang Z, Hong JS, Huang B, ^17^ despite meeting most of the inclusion criteria, was a clinical study that did not specify clearly if the extract was obtained from Morus alba leaves. Also, the research of Qi S, Li N, Tuo Z, Li J, Xing S, Li B, *et al*., ^18^ despite meeting most of the inclusion criteria, was excluded because the extract used did not specify if it had been made with Morus alba leaves. Finally, the research of Todd SB, Fallah S, Salemi Z, Seifi M. ^19^ was excluded because it measured the work, measured the level of specific hormones, but not that of the constituents of the Morus alba leaf that participate in the obesity.

## Discussion

The genus Morus includes several species, such as *Morus alba*, *M. nigra*, both native to Asia, and *M. rubra* of North American origin. With wide distribution in the world, this genus, both in temperate and tropical areas, reports various medicinal properties, one of the most studied being *Morus alba.*

A study based on the network approach or network pharmacology found that the alkaloids, flavonoids, and polysaccharides of *Morus alba* leaves impart regulatory effects on blood sugar levels, identifying 202 components, of which 22 can have significant curative effects on DM. ^20^ To study its main bioactive components such as anthocyanins, polysaccharides, phenols, alkaloids, and flavonoids, at the phytochemical level, various techniques have been used that include extraction and separation processes such as solid-liquid extraction, solid-phase extraction, pressurized liquid extraction, extraction by supercritical fluid, microwave-assisted extraction, ultrasonic-assisted extraction, enzyme assisted extraction, macroporous resin adsorption, silica gel chromatography, ion-exchange chromatography gel filtration chromatography, preparative liquid chromatography, and countercurrent chromatography. ^21^

Triterpenes and steroids are found in *Morus alba* leaf, fruit, and root extracts, as well as phenols and tannins and secondary metabolites with pharmacological properties for the treatment of many diseases associated with oxidative stress, such as Diabetes. ^22^ The leaves of *Morus alba* contain flavonoids as main constituents, which, in addition, possess various biological activities, including antioxidant, antidiabetic, antihyperlipidemic, antiobesity, cardioprotective activities, and are rich in anthocyanins and alkaloids. ^13^ Rutin or quercetin-3-rutinoside is a flavonoid glycoside obtained from the leaves of *Morus alba*, and its antiobesity effect has been verified by studying the lipid accumulation mechanism in 3T3-L1 adipocyte and C57BL mouse models. ^23^ Both compounds, rutin, and quercetin-3-O-β-D-glucoside (Q3G), have been shown to enhance glucose uptake through the AKT-mediated insulin signaling pathway or AMP-activated protein kinase activation (AMPK) in 3T3L1 adipocytes and could potentially be used in therapy for T2DM management. ^11^ In addition to rutin, the leaves of *Morus alba* contain apigenin, quercetin-3-triglyceride, lupeol, β-sitosterol, moracetin, isoquercitrin, coumarin, volatile oil, alkaloids, amino acids, and organic acids, proving the hypoglycemic properties and their effect enhancer on diabetic nephropathy. ^24^ Four main chemical components were identified in the ethanol extract of *Morus alba*: chlorogenic acid, rutin, isoquercitrin, and quercitrin. MLE was found to improve hyperglycemia, insulin resistance, and dyslipidemia in T2 DM rats with a significant therapeutic effect. ^25^

1-deoxynojirimycin (DNJ) is another compound present in the leaves of *Morus alba.* It is an effective inhibitor of α-glucosidase, providing a significant hypoglycemic effect. Its primary mechanism of action occurs after 1-deoxynojirimycin enters the human body, with the inhibition of sucrose, maltase, α-glucosidase enzyme, and α-amylase decomposing starch, so it can block the absorption of carbohydrates in the body, inhibiting the increase in blood sugar to achieve the effect of the prevention and treatment of DM. ^26^ DNJ controls postprandial hyperglycemia by acting as a natural inhibitor of α-glucosidase in patients with T2DM. ^27^

Experimental models and laboratory techniques that evaluate various pharmacological activities have been included in this review. Several investigators have used streptozotocin-induced Wistar rats for the empirical part, administering *Morus alba* leaf extract. One of them demonstrated antioxidant activity, and antidiabetic effect of ethanol extracts with high levels of phenolic acids, chlorogenic acid, and flavonol glycosides, finding efficacy in the correction of hyperglycemia in the increased secretion of insulin. Two other investigations corroborated the improvement of the antioxidant situation ^28^ and that administering high doses (0.25%) and low doses (0.5%) of *Morus alba* leaf tea demonstrated hypolipidemic, hypoglycemic, and improvement on diabetic nephropathy. ^24, 29^ Another study mentions that administering *Morus alba* ethanol extracts in doses of 0.5-1g/kg and oxyresveratrol (0.6 g/kg), an essential compound in the extract, could be considered as a potential adjunct therapy for the management of Diabetes since both reduced the level of glucose from fasting blood and plasma glucose. ^30^ Likewise, in another study, ethanolic extract of *Morus alba* leaves (100 mg/kg) was orally administered for eight weeks to female Wistar rats that were fed a high-cholesterol diet to evaluate the lowering effect of obesity, dyslipidemia, and insulin resistance, showing significant results since it decreased body weight gain, hypercholesterolemia, hypertriglyceridemia, atherogenic indices, based on lipid profile, and coronary artery, glucose level, and the insulin resistance index. ^31^ The effect of *Morus alba* leaf extract (FME) on insulin resistance through IRS-1 / PI3K / Glut-4 signaling pathway in rats with T2DM was also evaluated; the results obtained showed that the FME showed improvement in insulin resistance, in addition, a decrease in body weight, blood glucose, triglycerides, total cholesterol, and low-density lipoprotein levels were observed, significant antihyperglycemic and antihyperlipidemic activities via the pathway signaling IRS-1/PI3K/Glut-4, ^33^ Khyade (2018) obtained positive pharmacological effects on specimens of *Rattus norvegicus* induced with Diabetes, using a decoction of *Morus alba* leaves in doses of 20 g/L per day as treatment. The results found were decreased blood glucose levels and altered regulation of metabolic processes. ^32^

The effects of dietary supplementation with *Morus alba* leaf extracts with acetone-water (AE) and ethanol-water (EE) were also evaluated on the situation of the trace element (Fe, Zn, and Cu) about the management of Diabetes and antioxidant indices in diabetic rats fed the high-fat STZ diet and biochemical analyzes (glucose, thiobarbituric acid reactive substances, ferric reducing ability of plasma) were performed in blood serum, which showed that EA decreased the hepatic and renal storage of EF, while the EE increased the levels. ^33^ Laboratory tests were performed using mice induced to Diabetes and treatment with flavonoids extracted from the leaves of *Morus alba* on skeletal muscle cells, resulting in an improvement in mitochondrial function by activating AMPK in type 2 diabetes, improving resistance to the Skeletal muscle insulin significantly lowering blood glucose levels. ^34^

Likewise, it was investigated whether flavonoids, which are extracted from the leaves of *Morus alba*, can regulate glycolipid metabolism and to investigate whether flavonoids could regulate the signal of the IRS1 / PI3K / AKT pathway to affect the expression of FAS and GLUT4 membrane transfer capacity in 3T3. The results showed that the flavonoids from *Morus alba* leaf extracts alleviated the metabolic abnormalities of glycolipids in the IR model of 3T3-L1 adipocytes. The effect was associated with the activation of the IRS1/PI3K/AKT pathway. ^31^

A double-blind, randomized clinical trial was conducted in animals and in patients with glucose intolerance, who were administered a standardized extract to evaluate the antihyperglycemic effect, the inhibitory effect of α-glucosidase, the acute single oral toxicity, and the reduction of blood glucose in a test. The standardized extract of *Morus alba* leaves was found to inhibit α-glucosidase at a level four times higher than the positive control (acarbose), in a concentration-dependent manner, proving to be an excellent potential material to improve postprandial hyperglycemia. ^35^

Work has been carried out with the interaction of the leaves of *Morus alba* and other plants. One of them used fenugreek seed (Trigonella foenum-graecum), *Morus alba* Leaf, and American ginseng root (Panax quinquefolius) can improve glycemia in humans and animals with impaired glucose and T2DM metabolism, improving insulin sensitivity and glucose absorption in human adipocytes. ^36^ Another study evaluated treatment options for obesity, applying a standard composition (UP603), composed of extracts of *Morus alba*, *Ilex paraguariensis*, and *Rosmarinus Officinalis* in diet-induced obese C57BL/6J mice. UP603 treated animals showed decreased body weight gain, 65.5% and 16.4% reductions in insulin and leptin, respectively, and a 2.1-fold increase in ghrelin level. In addition, there were reductions from 7.9% to 21.1% in total cholesterol, from 25.4% to 44.6% in triglycerides, and from 22.5% to 38.2% in cholesterol linked to lipoproteins. Low-density lipoprotein (LDL) in mice treated with 450-850 mg/kg of UP603. ^37^

One method used to standardize the *Morus alba* extract is high-performance reversed-phase liquid chromatography (RP HPLC). The plant extract and the single bioactive molecule were studied for their potential for inhibition of combined CYP450, as well as forms of recombinant human CYP450, such as the isoenzymes CYP3A4, CYP2D6, CYP2C9, and CYP1A2, the findings suggested that the plant extract and its bioactive compound are safe to use in the management of Diabetes. ^38^

Likewise, experimental studies in diabetic rats and normal rats have shown that the fruit and bark of the *Morus alba* root can potentially be used as an effective treatment for DM2 ^39^ and effectively improve hyperlipidemia and inhibit diacylglycerol acyltransferase. ^18^

A systematic review indicates that several studies have shown that the aqueous extracts of the bark of roots, leaves, and stems contain polyphenols and polysaccharides with antihyperglycemic and antihyperlipidemic properties; polysaccharides have been further studied to assess their antidiabetic bioactivity in T2DM-induced rats, clarifying the underlying mechanism of these activities. ^9^

A systematic review is presented on the chemical composition of the different parts of *Morus alba* and its pharmacological effects on DM, which include the influence on glucose absorption, production on insulin secretion as an antioxidant and anti-inflammatory agent. The study found that *Morus alba* leaf contains steroids and triterpene compounds, flavonoids, coumarin, essential oils, amino acids, alkaloids, and organic acids. The branch of *Morus alba*, for its part, contains tannin, fructose, stachyose, glucose, maltose, honeydew, and arabinose. The bark of the *Morus alba* root contains flavonoids, such as mulberry, mulberrochromene, and cyclomulberrine. The different parts of the plant contain active principles such as Polyhedral alkaloids, polysaccharides, and flavonoids are components that are found in all aspects of the *Morus alba* plant; the fruits also contain vitamin B1, B2, and carotene, also, fatty acids (linoleic, oleic and stearic). ^10^

Another review to evaluate the potential of *Morus alba* as a natural agent in the treatment of obesity found that the inhibitory effects of *Morus alba* on digestive enzymes and adipocyte differentiation and its stimulating effects on energy expenditure and Lipid metabolism are mechanisms responsible for the management of obesity in obese patients. ^40^

## Conclusions

It has been shown that the aqueous extracts of the leaves of *Morus alba* at a phytochemical level contain 202 constituents, among them routine with proven antiobesity effect, rutin together with quercetin-3-O-β-D-glucoside (Q3G) improve the glucose uptake and demonstrated antioxidant activity and antidiabetic effect.

The compound in the *Morus alba* leaf, 1-deoxynojirimycin (DNJ), has been identified as an effective inhibitor of α-glucosidase and provides a significant hypoglycemic effect, which could improve postprandial hyperglycemia and also prevent and treat in DM, oxyresveratrol could be considered as a potential adjunct mechanism for the management of Diabetes.

Chlorogenic acid, routine, isoquercitrin, and quercitrin present in *Morus alba* leaves have been shown to have hypoglycemic properties and an ameliorating effect on diabetic nephropathy, insulin resistance, and dyslipidemia in rats.

Various systematic reviews have evaluated that *Morus alba* leaves have various pharmacological effects on T2DM, including glucose absorption, production in insulin secretion, antioxidant and anti-inflammatory agent, antihyperglycemic and antihyperlipidemic activities, and obesity management.

The results of this research have a theoretical and scientific relevance because it manages to systematize scientific research relevant to the cognitive needs that the need for the use of *Morus alba* leaf for the treatment of type 2 diabetes mellitus raises today, which, as explained in this research, is a public health problem worldwide.

## Limitations

The results achieved allow identifying a lack of studies on the potential and synergistic effects of the components of the *Morus alba* leaves; this limits the possibility of a more effective therapy using the leaves of the plant; Furthermore, there are few studies on the extraction methodology of the components of the *Morus alba* leaf. These limitations must be taken into account for the orientation and development of new research.

## Recommendations

T2DM and the risk factors involved in the appearance of aggravating conditions of this disease, it should be considered that the extracts of the leaves of *Morus alba* constitute an effective alternative to keep glycemia and lipidemia under control in patients with T2DM, being an excellent complement to your usual treatment. It would also be necessary to expand pharmacological safety studies and preclinical and clinical studies of the use of *Morus alba* leaf extracts to generate more scientific evidence, which allows new forms of administration to be known.

## Data availability

### Underling data

All data underlying the results are available as part of the article and no additional source data are required.

### Extended data

Zenodo: Medicinal properties of Morus alba for the control of type 2 diabetes mellitus: a systematic review. https://doi.org/10.5281/zenodo.5227684. ^41^

This project contains the following extended data:

• Search Strategy.pdf
• Flow diagram and included articles.pdf
• Observation Guide (Quality Assessment of Case-Control Studies).pdf
• Observation Guide (Quality Assessment of Systematic Reviews and Meta-Analyses).pdf
• Scale to Evaluate Scientific Articles in Social and Human Sciences- SSAHS.pdf
• Prospero Registration.pdf

### Reporting guidelines

Zenodo: PRISMA checklist for ‘Medicinal properties of Morus alba for the control of type 2 diabetes mellitus: a systematic review’. https://doi.org/10.5281/zenodo.5227684. ^41^

Data are available under the terms of the Creative Commons Attribution 4.0 International license (CC-BY 4.0).
