# Supplementary material for: Medicinal properties of Morus alba for the control of type 2 diabetes mellitus: a systematic review
Source: F1000Res. 2021 Oct 8;10:1022. [Version 1] doi: 10.12688/f1000research.55573.1 (PMC8593624; doi:10.12688/f1000research.55573.1)
Supplement: Peer review history

# Peer review: Reviewer response for version 1

**DOI:** 10.5256/f1000research.59162.r97611

**Published:** 2021-11-15

**Authors:** Miryam Griselda Lora Loza

**Affiliations:** 1 Universidad Cesar Vallejo, Trujillo, Peru

The article belongs to the research line of Biomedical Sciences, specifically phytochemistry and pharmacognosy in medicinal plants, addressing the evidence of the activity of a plant on Diabetes Mellitus 2. It is a relevant and current topic in the medical specialty; the study developed a careful screening based on the various sources of articles consulted and included in the study.

 Some recommendations:

1. In the abstract –methodology section–, the wording should be improved so that it is clearly understood which studies were found, and of the total, which ones were included.
2. Review the publication “Declaración PRISMA 2020: una guía actualizada para la publicación de revisiones sistemáticas;” especially figure 1, page 795 ^1^ compare it with figure 1 of the manuscript under review (Flow diagram of the identification, screening, eligibility and included articles), and make the appropriate adjustments.
3. Synthesize the conclusion, focusing mainly on the study's main findings and their impact on the therapeutic potential of *Morus alba* leaves in the control of the disease addressed in the present study. 

Are the rationale for, and objectives of, the Systematic Review clearly stated?

Yes

Is the statistical analysis and its interpretation appropriate?

Partly

Are sufficient details of the methods and analysis provided to allow replication by others?

Yes

Are the conclusions drawn adequately supported by the results presented in the review?

Partly

Reviewer Expertise:

Health & Wellness ; Public Health

I confirm that I have read this submission and believe that I have an appropriate level of expertise to confirm that it is of an acceptable scientific standard.

## References

[1] : Declaración PRISMA 2020: una guía actualizada para la publicación de revisiones sistemáticas. *Revista Española de Cardiología* .2021;74(9) : 10.1016/j.recesp.2021.06.016 790-799 10.1016/j.recesp.2021.06.016 34446261

# Peer review: Reviewer response for version 1

**DOI:** 10.5256/f1000research.59162.r96495

**Published:** 2021-11-09

**Authors:** José Enrique Alfonso Manzanet

**Affiliations:** 1 Centro Nacional de Información de Ciencias Médicas, Havana, Cuba; 2 Universidad de Ciencias Médicas de La Habana, La Habana, Cuba

The study carries out a detailed analysis of the selected articles. The information search criteria are adequate and the results are correctly analyzed. However, it is recommended to be more explicit in the limitations of the study in question. It cannot be said that it effectively constitutes an effective treatment alternative when selected studies have been carried out in rats. I think the recommendations are aimed at expanding the clinical study in humans.

Are the rationale for, and objectives of, the Systematic Review clearly stated?

Yes

Is the statistical analysis and its interpretation appropriate?

Not applicable

Are sufficient details of the methods and analysis provided to allow replication by others?

Yes

Are the conclusions drawn adequately supported by the results presented in the review?

Yes

Reviewer Expertise:

Scientific Comunication; Pharmacology

I confirm that I have read this submission and believe that I have an appropriate level of expertise to confirm that it is of an acceptable scientific standard.
